# Influence of age on rat bone-marrow mesenchymal stem cells potential

**DOI:** 10.1038/srep16765

**Published:** 2015-11-19

**Authors:** J. Fafián-Labora, P. Fernández-Pernas, I. Fuentes, J. De Toro, N. Oreiro, S. Sangiao-Alvarellos, J. Mateos, M.C. Arufe

**Affiliations:** 1 Grupo de Terapia Celular y Medicina Regenerativa (TCMR-CHUAC). CIBER-BBN/ISCIII. Servicio de Reumatología. Instituto de Investigación Biomédica de A Coruña (INIBIC). Complexo Hospitalario Universitario de A Coruña (CHUAC). SERGAS. Departamento de Medicina. Facultade de Oza. Universidade de A Coruña (UDC). As Xubias, 15006. A Coruña, Spain; 2 Grupo de Proteómica-PBR2-ProteoRed/ISCIII-Servicio de Reumatologia. Instituto de Investigación Biomédica de A Coruña (INIBIC), Complexo Hospitalario Universitario de A Coruña (CHUAC), Sergas. Universidade da Coruña (UDC). As Xubias, 15006. A Coruña, España; 3 Grupo Fisiopatología Endocrina, Nutricional y Médica (FENM-CHUAC). Instituto de Investigación Biomédica de A Coruña (INIBIC). Complexo Hospitalario Universitario de A Coruña (CHUAC). SERGAS. Departamento de Medicina. Facultade de Oza. Universidade de A Coruña (UDC). As Xubias, 15006. A Coruña, Spain

## Abstract

Mesenchymal stem cells promising role in cell-based therapies and tissue engineering appears to be limited due to a decline of their regenerative potential with increasing donor age. Six age groups from bone marrow mesenchymal stem cells of Wistar rats were studied (newborn, infant, young, pre-pubertal, pubertal and adult). Quantitative proteomic assay was performance by iTRAQ using an 8-plex iTRAQ labeling and the proteins differentially expressed were grouped in pluripotency, proliferative and metabolism processes. Proliferation makers, CD117 and Ki67 were measure by flow cytometry assay. Real time polymerase chain reaction analysis of pluripotency markers Rex1, Oct4, Sox2 and Nanog were done. Biological differentiation was realized using specific mediums for 14 days to induce osteogenesis, adipogenesis or chondrogenesis and immunostain analysis of differentiated cell resulting were done. Enzimoimmunoassay analysis of several enzymes as L-lactate dehydrogenase and glucose-6-phosphate isomerase were also done to validate iTRAQ data. Taking together these results indicate for the first time that mesenchymal stem cells have significant differences in their proliferative, pluripotency and metabolism profiles and those differences are age depending.

Mesenchymal Stem Cells (MSCs) have self-renewal capacity and multiple differentiation potentials, and *a priori*, could play important roles in regenerative medicine. The promising role of MSCs in cell-based therapies is their trophic, paracrine and immunomodulatory functions that may have the greatest therapeutic impact *in vivo*^1^,^2^. Tissue engineering from MSCs are of highly importance for regeneration of mesenchymal tissues such as craniofacial bone^3^, cartilage^4^ and connective tissues^5^.

Since several years ago, numerous studies have shown that MSCs from different tissues have similar levels of surface antigen expression, immunosuppressive activity, and differentiation ability^6^. The ability of MSC to carry out normal tissue regeneration in the body and their potential for using in clinical applications may be impaired by loss of stem cell number and function with age^7^. There are different rate of cell proliferation and clonality between MSCs depending of source from the cells are obtained^8^ and the chronological age form the donors and also the number of the *in vitro* culture passages^9^. MSCs are missing their characteristics during the chronological or *in vitro* culture of them^10^ but not in the same way. Various attempts have been made to address challenges associated with aging of MSC including culture in hypoxic conditions^11^ and ectopic expression of pluripotency-associated factors^12^. This study applied the 8-plex iTRAQ system to analyze MSCs from six different aging groups, as this quantitative proteomic technology has the capability to compare several time points in a single experiment.

The major contributor to the development of the senescent cellular phenotype is hyper activation of nutrient sensor and growth pathways, in particular mTOR and its derivative complexes mTORC1 and mTORC2^13^,^14^. mTOR family regulates senescence and autophagy during reprogramming of somatic cells to pluripotency indicating the important role of energy metabolism to stem cell renewal and aging^15^. We studied the relationship between mTOR and the proliferation markers CD117 and Ki67 using imatinib mesylate, the inhibitor of tyrosine kinase receptor for CD117^16^ and JK184 which reduce expression of Ki67^17^. We establish a framework for future comparative and functional studies; we have analyzed the phenotypic, genotypic features and biological-related changes in MSCs of rat bone marrow from animals of different ages.

## Material and Methods

### Isolation and culture of cells

For isolation of MSCs, the animals were anesthetized with Fluorane (Izasa, A Coruña, SP) and sacrificed by cervical dislocation method. Femurs were dissected from male Wistar rat (Animal Service, CHUAC) at different ages: neonate (0 days old), infant (7 days old), young (14 days old), pre-pubertal (35–38 days old), pubertal (45 days old) and adult (2 months old). All the methods were carried out in “accordance” with the approved guidelines of Spanish law (32/2007). All experimental protocols were approved by Animal Ethical Committee of Galicia. The protocol used by Karaoz *et al.*^18^ was followed in this work. Briefly, the ends of the bones were cut away and a 21-gauge needle that was inserted into shaft of the bone marrow was extruded by flushing with 5 ml D-Hank’s solution supplemented with 100 IU/ml penicillin–100 mg/ml streptomycin (all from Life Technologies, Madrid, Spain). Marrow plug suspension was dispersed by pipetting, successively filtered through 70-μm mesh nylon filter (BD Biosciences, Bedford, MA, USA), and centrifuged at 20000 g for 10 min. Supernatant containing thrombocytes and erythrocytes was discarded, and the cell pellet was resuspended in the medium. The cells from four rats were seeded onto 100 cm^2^ dish plate (TM Nunclon) and incubated at 37 °C with 5% humidified CO_2_. The MSCs were isolated on the basis of their ability to adhere to the culture plates. On the third day, red blood cells and other non-adherent cells were removed and fresh medium was added to allow further growth. The adherent cells grown to 70% confluence were defined as passage zero (P0) cells. After 5 min of centrifugation, 1 × 10^6^ MSCs were seeded on two dish plates 100 cm2 (TM Nunclon) in RPMI supplemented with 10% fetal bovine serum (FBS), 100 U/ml penicillin and 100 mg/ml streptomycin (all from Sigma-Aldrich, St. Louis, MO, USA). The medium was added and replaced every 3 or 4 days for 2 weeks. Before being used in inducing mesoderm differentiation, the MSCs had been expanded for 2 passages and characterized. Afterwards MSCs from adult group were incubated during 12 hours previously to protein extraction with imatinib mesylate or JK184 (all from Sigma-Aldrich).

### Flow Cytometry

To characterize the different populations of MSCs from chronological different animals, their MSCs were washes twice in PBS (Sigma-Aldrich, St. Louis, MO, USA), then pre-blocked with 2% rat serum in PBS. The following direct antibodies were used: PE-conjugated mouse anti-human CD34 (1:20 from DakoCytomation, Barcelona, SP); FITC-conjugated mouse anti-rat CD45 (1:20 BD Pharmingen, New Jersey, USA); PE-Cy5.5-conjugated mouse anti-rat CD90 (1:20 Immunostep, Salamanca, SP) and APC-conjugated mouse anti-rat CD29 (1:20 Immunostep, Salamanca, SP). The cells were washed with PBS after one hour of incubation with the corresponding antibody at room temperature. To check proliferation profile of the different populations of MSCs from chronological different animals, their MSCs were incubated with APC conjugated mouse anti-rat CD117 (1:20 Immunostep, Salamanca, SP), mouse anti-BRDU (sigma-Aldrich) and FITC conjugated mouse anti-human Ki67 (1:20 Immunostep, Salamanca, SP). The secondary FITC-conjugated rabbit anti-mouse antibody was used to link cells incubated with anti-BRDU. The stained cells were then washed twice with PBS and 2 × 10^5^ cells were analyzed with a FACSAria flow cytometer (BD Science, Madrid, SP). FACS data was generated by DIVA software (BD Science). Negative control staining was performed using FITC-conjugated mouse IgG1K isotype, PE- conjugated mouse IgG1K isotype, PE-Cy5.5- conjugated mouse IgG1K isotype and APC- conjugated mouse IgG1K isotype (all from BD Pharmingen). For the intracellular ROS accumulation was used H_2_DCFDA. Upon oxidation by ROS, the non-fluorescent H_2_DCFDA is converted to the highly fluorescent 2′,7′-dichlorofluorescein. MitoSOX™ Red Reagent was used to determine mitochondrial ROS including superoxide dismutase activity. Tetramethylrhodamine, methyl ester (TMRM) (from Thermo Fisher Scientific, Life Technologies, SP), the permanent dye that accumulates in active mitochondria with intact potentials, was used to detect functional mitochondria in the MSCs at different ages following functional mitochondrial staining protocol from commercial.

### Proliferation assay

Different numbers of cells (0, 1000, 2000, 4000, 8000 and 16000 cells), were plated in triplicate in 96-well plates and allowed to adhere for 8 hours, were used to calculate the proliferation curve. The number of cells was then calculated using CellTiter 96® AQueous Non-Radioactive Cell Proliferation Assay (Promega, Madison, WI, USA) following manufacturer instructions. For the assay, 4000 cells were plated for each cell line in triplicate in 96-well plates, and the total number of cells was calculated at different time points (0, 1, 2, 5 and 6 days).

The cytotoxicity assay was realized using Cell Counting Kit-8 (Dojindo Molecular Technologies, Inc. MD 20850, USA). Briefly 100 all of cell suspension (5000 cells/well) in a 96-well plate were incubated for 24 hours at at 37 °C, 5% CO_2_. After that medium with imatinib mesylate or JK184 was added to the wells 48 hours later, 10 μl of CCK-8 solution was added to each well of the plate. After four hours incubation the absorbance was measured at 450 nm using a microplate reader.

### Histological and immunohistochemical analysis

MSCs following differentiation into chondrocyte-like or adipocyte-like cells were frozen in OCT embedding matrix (BDH Chemicals, Poole, UK). The cells were fixed in 4% (w/v) paraformaldehyde (Sigma-Aldrich) in PBS at pH 7.6. Some cells were stained with Safranin O, Modified Masson´s (Sigma-Aldrich) to evaluate the distribution of proteoglycan in the extracellular matrix generated by the cells differentiated towards chondrocyte-like cells. Other cells were stained with Alizarin red (Sigma-Aldrich) to check alkaline deposits in cells differentiated towards osteocyte-like cells. Other cells were stained with Oil red (Sigma-Aldrich) to check oil drops in cells differentiated towards adipocyte-like cells.

### Densitometry analysis

AnalySIS Image Processing (Soft Imaging system GmbH V. 5.0, Olympus, Münster, Germany) was used to do a densitometry quantification of the staining obtained by immunohistochemistry analysis shown in the plots. Three fields 200 mm^2^ in size from each inmunostain- safranine O, oil red, modified Masson’s and alizarin red - and time studied were quantified using arbitrary units for immunohistochemistry values provided by the computer program. Values expressed as percentage of positive stain for each marker studied were used for immunohistochemistry analysis. All values were referenced with respect to values obtained from cells cultured in the control medium (RPMI 5% knockout serum, 1% penicillin and 1% streptomycin).

### Real time quantitative polymerase chain reaction (qRT-PCR) analysis

Primers for amplification of rat Rex1, Oct4, Sox2 and Nanog genes were used to determine the expression of those markers for pluripotency in the different populations of MSCs from chronological different animals. Details are shown in Table 1. The amplification program consisted of initial denaturation at 92 °C for 2 minutes followed by 40 cycles from 92 °C for 15 seconds, annealing at 55–62 °C, depending on the gene, for 30 seconds and extension at 72 °C for 15 seconds. PCR analyses were done in triplicate, with each set of assays repeated three times. To minimize the effects of unequal quantities of starting RNA and to eliminate potential sources of inconsistency, relative expression levels of each gene was normalized to ribosomal protein (HPRT) using the 2-ΔΔCt method^19^. Control experiments utilized no reverse transcriptase.

### Protein isolation and immunoblot analysis

Immunoblot analysis was performed on 40 μg of total protein extracted from MSCs, as previously described^20^. The blots were probed with antibodies, made into rabbit, directed against mTOR, raptor (Cell Signaling, Izasa, Madrid, ES), vimentin, superoxide dismutase (SOD-2), lamin A/C and tubulin (all form Sigma-Aldrich) was used for housekeeping. A secondary anti-goat antibody (Cell Signaling) was used to visualize proteins using an Amersham^TM^ ECL^TM^ Western Blotting Analysis System (GE Healthcare, Amersham Biotechnology, Manchester, UK). Ideal concentrations for each antibody were determined empirically. Working concentrations were 1:1000 of the recommended stock solutions.

### iTRAQ labelling

Equal amounts of proteins from each group of different age cells (100 μg) were denatured with 2% sodium dodecyl sulfate (SDS) in 1 M tryethylammonium bicarbonate (TEAB) (ABSciex, Foster City, CA). The samples were then reduced for 1 h at 60 °C using 50 mM tris-(2-carboxyethy) phosphine (TCEP) (ABSciex), and cysteine-blocked with 84 mM iodoacetamide (Sigma-Aldrich) at room temperature in the dark for 30 min. The proteins were digested with spectrometry grade trypsin (Gold Mass, Promega, Madison,WI) at a concentration of 1:50 trypsin/protein for 16 h at 37 °C. Each peptide solution was labeled for 1.5 h at room temperature using the iTRAQ reagents previously reconstituted in 70 μL of ethanol, following the manufacture Protocol (ABSciex). The samples were labeled with iTRAQ reagents as follows: newborn: 119 and 121 as a control infant: 114; young: 116; pre-pubertal: 118; pubertal: 115; adult: 117. The reaction was stopped by adding deionized water, and the labeled samples were combined. The mixture was desalted using home-made stage-tips.

### iTRAQ relative quantification by 2D-LC-MALDI-TOF/TOF analysis

In a first step, the desalted peptides were fractionated by basic reversed phase extraction in a 1400 HPLC system (Agilent). The fractions were collected along a 110 minutes gradient and subjected to further acidic reversed phase extraction in a nanoHPLC system (Tempo, ABSciex) into a C18 silica-based column (New Objective, Woburn, MA) with an internal diameter of 300 Ả. The injection volume was 5 μL, and peptides were eluted during a ninety minutes gradient at a constant flow rate of 0.35 μL/min. Eluting peptides were automatically mixed with alpha-cyano at 4 mg/mL en 70% AcN, TFA 0.1% and deposited on a MALDI LC-plate using a SunCollect (SunChrom) spotter. The chromatograms, composed by 350 spots, each one comprising a 15 sec deposition, were then analyzed in a 4800 MALDI-TOF/TOF platform (ABSciex). 4000 series Explorer v.4.2 software was used to generate the spectra and peak list. After manual deposition of mass calibrates, plate model and default calibration of the MALDI plate was done with a laser voltage of 3200 kV and 1000 shots/spectrum. Samples were automatically analyzed in MS mode with a laser voltage of 3400 kV and 1500 shots/spectrum. Automated precursor selection was done using a Job-wide interpretation method (up to 12 precursors/fraction, Signal to Noise lower threshold = 50) excluding trypsin autolytic peptides and other background ions, with a laser voltage of 4200 and 2000 shots/spectrum. CID collision energy range: medium. LC-MALDI-TOF/TOF data were analyzed using ProteinPilot 4.0 software (ABSciex). Protein Pilot Search parameters were as follows: Sample type: iTRAQ 8-plex; Cys-alkylation: iodoacetamide; Digestion: trypsin; ID focus: Biological modifications; Database: last SwissProt release; Species filtering: none; Search effort: Thorough ID and Detection Protein Threshold Unused ProtScore (Conf)>1.3 (95.0%). Scoring model was defined by the Paragon algorithm. In the case of the high complexity samples, False Discovery Rate -FDR- was estimated in less than 1% by doing the searching in parallel against a decoy database using “PSPEP on” mode -data not shown-.

### Bioinformatics

Biological functional analysis of different modulated proteins detected by iTRAQ quantification, were categorized according to their function, biological process and cellular component, using the String 9.0 software^21^. Proteins with statistically significant changes were identified by filtering according to these criteria: 1) they had to be present in two biological replicates; 2) changes between groups had to be statistically significant (P < 0.05); and 3) fold change had to be greater than 1.2 and lower than 0.8 (date do not shown) This approach allowed us to select 201 differentially expressed proteins for further analysis.

### Enzymatic Analysis

5 × 10^5^ cells from each group of different age were used for the assessment of enzyme activities. The cells were homogenized in 200 μL of 250 mM sucrose(Sigma-Aldrich, St.Louis, MO), 50 mM HEPES (Sigma-Aldrich), 0,5 mM EDTA (Sigma Aldrich) and one tablet protease inhibitor cocktail (Roche, Mannheim, Germany ). Enzymes activities were determined using a SUNRISE spectrophotometer (TECAN, Mannedorf, Switzerland). Reaction rates of enzymes were determined by the increase or decrease in absorbance of NAD(P)H (Sigma-Aldrich, St.Louis, MO) at 340 nm at 37 °C. Lactate dehydrogenise (EC 1.1.1.27) was determined in BM-MSCs using 50 mM Trizma base (pH 7,4), 0,15 mM NADH and 5 mM sodium pyruvate (omitted for control) (all Sigma Aldrich, St.Louis, MO). Glucose-6-phosphate 1-deshydrogenase (EC 1.1.1.49) and 6-phosphogluconate dehydrogenase, decarboxylating (EC 1.1.1.343) was determined in BM-MSCs using 78 mM Trizma base, 5 mM MgCl2(pH 7,4), 0,1 mM NADP, 0,5 mM D-Glucose 6-phosphate disodium salt hydrate and 6-Phosphogluconic acid trisodium salt (omitted for control) (all Sigma-Aldrich, St.Louis, MO).

### Statistics

All experiments were performed in triplicate and one representative is shown. Non-parametric statistical analyses were performed by Mann-Whitney-U and Kruskal-Wallis tests using GraphPad Prism6 (GraphPad Software, La Jolla, CA). Each group was compared with previous group. A *p* value less than 0.05 or 0.01 were considered statistically significant. All the the data are presented as standard error of the mean.

## Results

**Figure 1:**
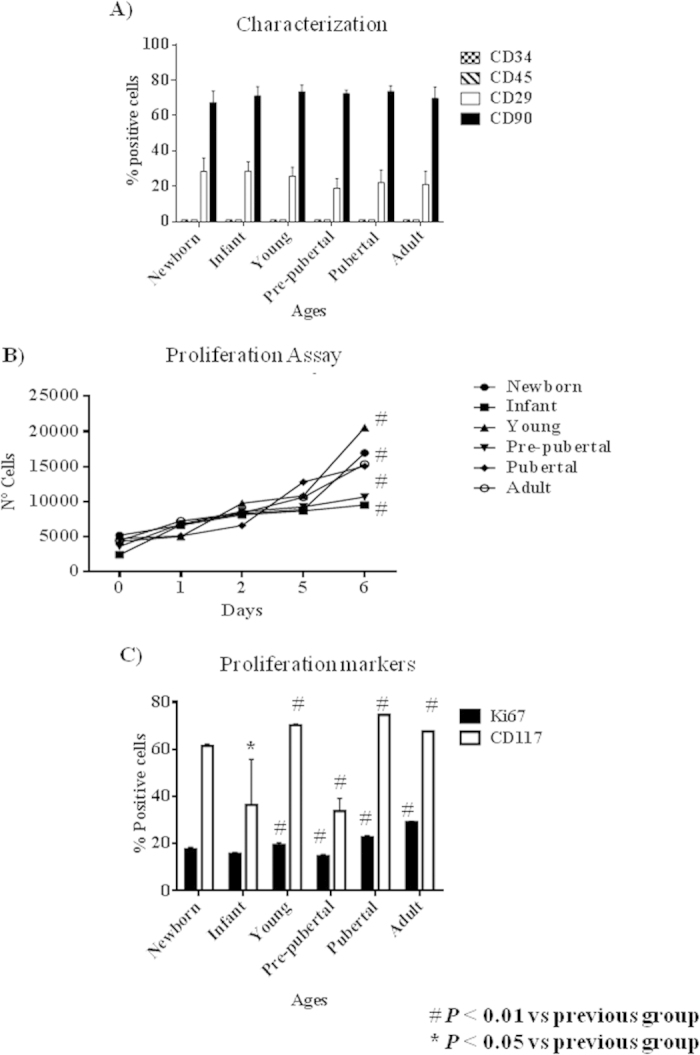
Proliferation profile from rat mesenchymal stem cells at different age. (**A**) Characterization by flow cytometry assay of percentage of positives mesenchymal stem cells markers (CD29 and CD73) and negative hematopoietic markers (CD34 and CD45). (**B**) Proliferation assay of studied aging groups for 6 days. (**C**) Percentage of proliferation markers, DC117 and Ki67, from studied aging groups by flow cytometry assay. One representative experiment is shown. ^#^*p* value less than 0.05 compared with previous group and *p value less than 0.01 compared with previous group, were considered statistically significant using Mann-Whitney-U tests.

Characterization of populations of MSCs from different ageing group by flow cytometry reveled that no statistical significant differences exist between group respects levels of mesenchymal and hematopoietic markers used for that (Fig. 1A). Positive cells for CD45 and CD34 were less than 1%, positive cells for CD29 were 30 ± 5% and positive cells for CD90 were 75 ± 5% in all groups studied.

Proliferation Assays results indicated that MSCs from groups of newborn (17 × 10^3^ ± 100), young (21 × 10^3^ ± 200), pubertal (15 × 10^3^ ± 300) and adult (16 × 10^3^ ± 100) animals had a statistically significant higher (p < 0.01) number the cells compared to infant (9 × 10^3^ ± 500), and pre-pubertal (10 × 10^3^ ± 500) (Fig. 1B).

Flow cytometry assays to detect CD117 and Ki67 positive cells indicated that MSCs from pubertal and young groups had the statistical significant (p < 0.01 and p < 0.05 respectively)) higher CD117 positive cells percentage of MSCs (74.65 ± 0.07 and 71.95 ± 3.10 respectively) than the rest of the groups studied, newborn: 61.53 ± 0.37; infant: 60.50 ± 1.58; adult: 61.12 ± 6.35 and pre-pubertal: 35.25 ± 2.14. On the other hand, infant and pre-pubertal groups had the statistical significant (p < 0.05) lower Ki67 positive cells percentage (15.63 ± 0.24 and 14.65 ± 0.41 respectively) than the rest of the groups studied, newborn: 18 ± 0.55; young: 19.33 ± 0.43; pubertal 22.68 ± 0.40 and adult: 29.02 ± 0.16 (Fig. 1C)

**Figure 2:**
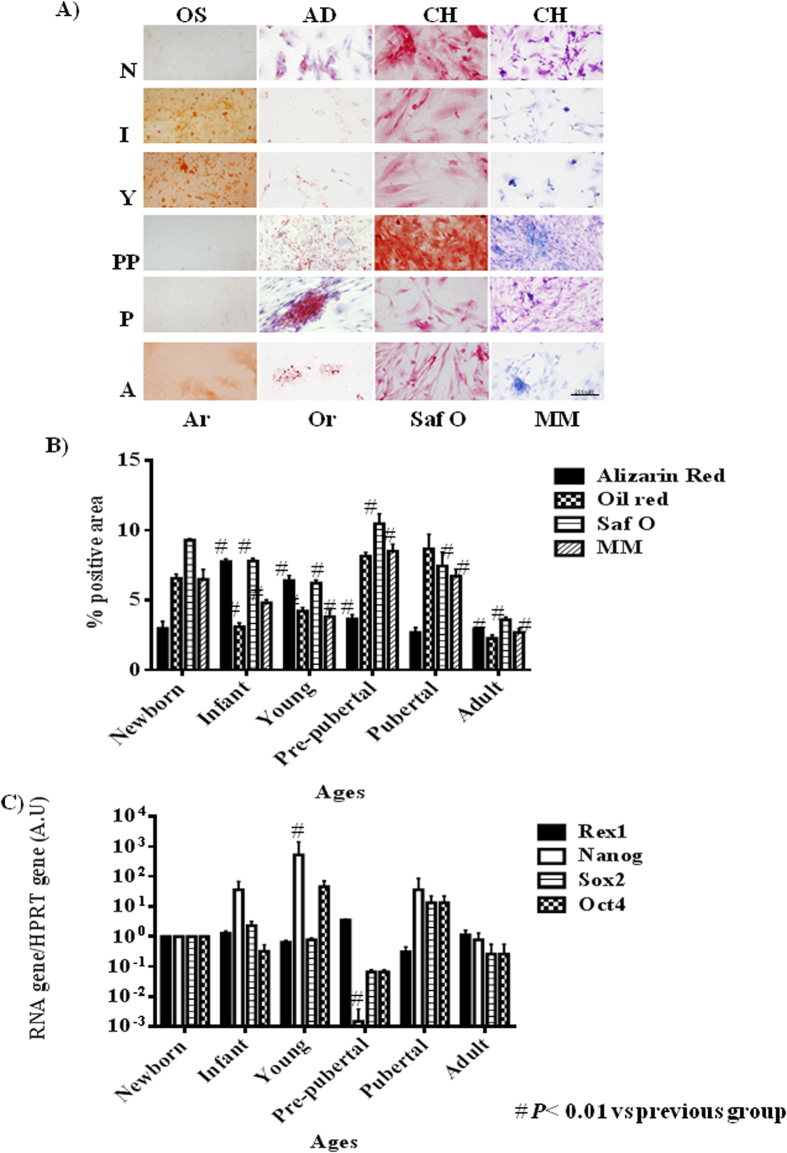
Pluripotency profile from rats mesenchymal stem cells at different age. (**A**) Representative pictures of mesenchymal stem cell from bone marrow of studied age group after 14 days with specific differentiation medium. On the top specific medium is indicated; OS=osteogenic medium; AD = adipogenic medium; CH = chondrogenic medium. Differentiation medium are indicated in Material and methods. On the bottom stain is indicated; Ar = alizarin red; Or = oil red; Saf O = safranine O and MM = Modified Masson’s stain. Straight size is 200 μM. (**B**) Densitometry study of mesenchymal stem cell from bone marrow of studied aging group after 14 days with specific differentiation medium after immunostaining assay. AnalySIS Image Processing computer was used to quantify the signal of different stain obtained. ^#^*p* value less than 0.01 was considered statistically significant using Mann-Whitney-U tests. (**C**) Histogram represents gene expression of pluripotency markers, Rex1, Nanog, Sox2 and Oct4. Real-time reverse transcriptase PCR (qRT-PCR) analysis normalized by expression of HPRT gene used as housekeeping. ^#^*p* value less than 0.01 compared with previous group was considered statistically significant using Mann-Whitney-U tests. Three replicates were made.

Differentiation capacity of the groups studied was tested through direct mesoderm induction using specific culture medium. It was observed that pre-pubertal group presented statistically significant (p < 0.05) highest stain for safranine O, modified Masson´s and oil red by histological analysis followed by pubertal with respect to the other groups. Young group presented the highest staining, statistically significant (p < 0.05) for alizarin red with respect to others groups and the adult group presented the lowest statistically significant (p < 0.05) differentiation potential with respect to other groups (Fig. 2A,B). Nanog, Oct4, Sox2 and Rex1 gene expression were tested by qRT-PCR analysis to check the pluripotency potential of the studied groups. The results shown the statistically significant (p < 0.05) highest expression of Nanog in young group with respect to the others groups in opposition of statistically significant (p < 0.01) decrease expression of this same gene, Nanog, in the pre-pubertal group respect to the others (Fig. 2C).

**Figure 3:**
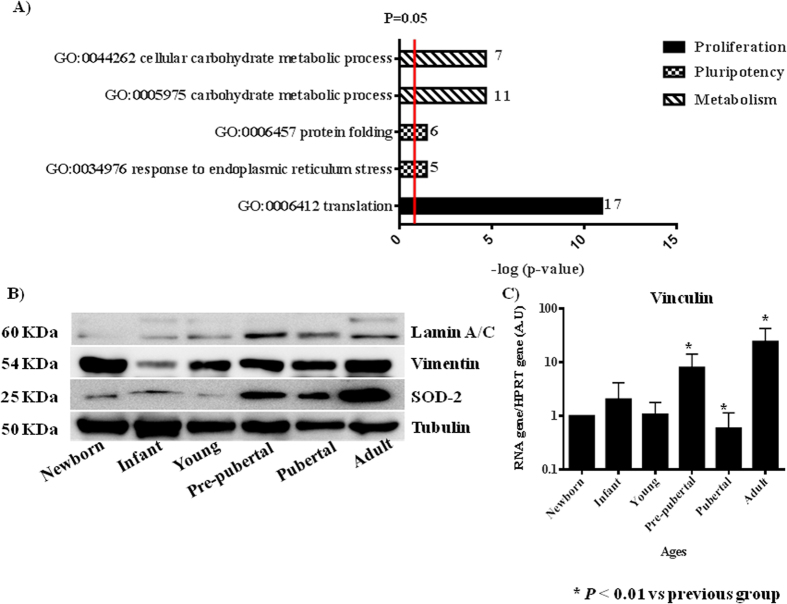
Validation of iTRAQ analysis. (**A**) Significant activates pathways obtained by comparing modulated proteins obtained by iTRAQ analysis employing functional annotations according to the String 9.0 software. Small numbers on the right of each bar are the modulated protein involved in each process. (**B**) Western blot analysis of Lamin A/C, Vimentin and Superoxide dismutase 2 (SOD-2). Tubulin was used as housekeeping. On the left molecular weight of each protein is shown. On the bottom the group’s source of mesenchymal stem cells used. The gels have been run under the same experimental conditions. (**C**) Vinculin gene expression using real-time reverse transcriptase PCR (qRT-PCR) analysis normalized by expression of HPRT gene used as housekeeping.

**Figure 4:**
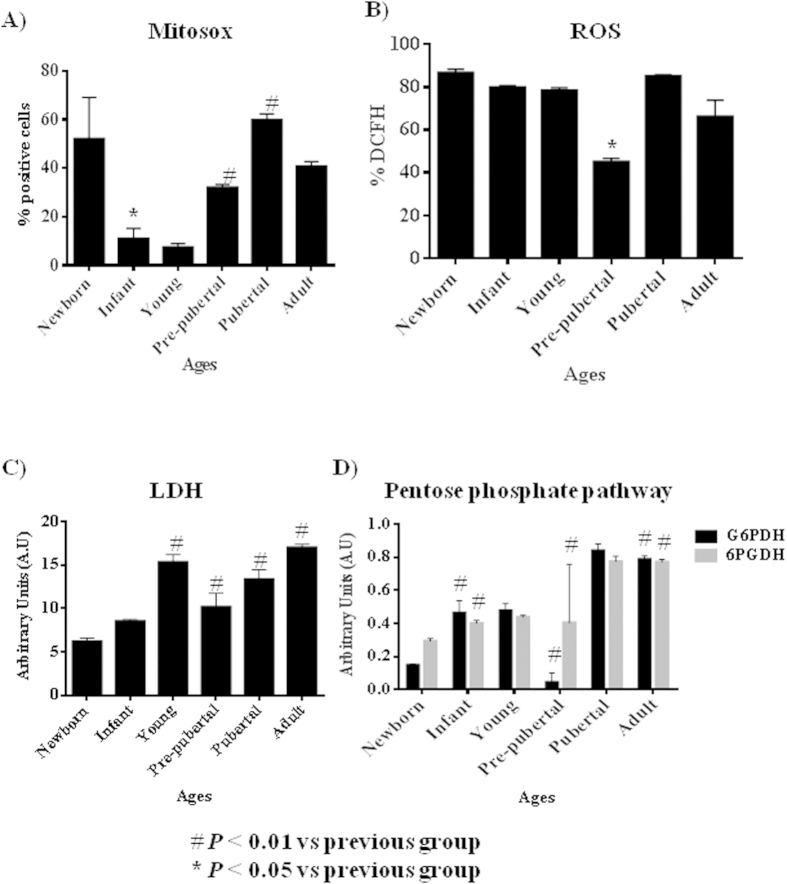
Metabolism profile from rats mesenchymal stem cells at different age. (**A**) 2′,7′-dichlorofluorescein signal measured by flow cytometry to check ROS intracellular (**B**) Mitosox signal measured by flow cytometry to check ROS mitochondrial. (**C**) Lactate-dehydrogenase (LDH) activity measured by spectrophotometer analysis. (**D**) Pentose phosphate pathway activity measured by spectrometer analysis. G6PDH = Glucose-6-phosphate 1-dehydrogenase; 6GPDH = 6-phosphogluconate-dehydrogenase. *p value less than 0.01 compared with previous group and ^#^*p* value less than 0.05 compared with previous group, were considered statistically significant using Mann-Whitney-U tests.

**Figure 5:**
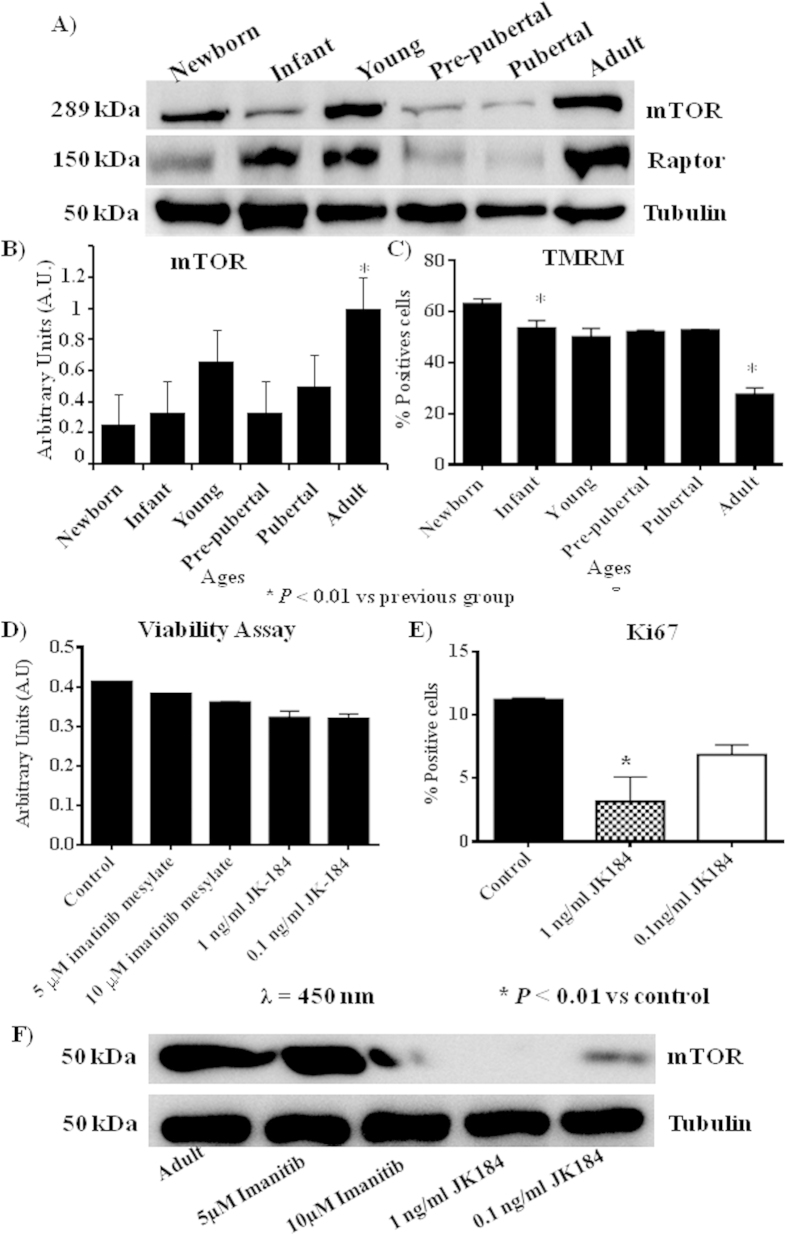
mTOR pathway profile from rat mesenchymal stem cells at different age (A) Western blot of mTOR pathway, mTOR and raptor, Tubulin was used as housekeeping. (**B**) Densitometry analysis of westerns of mTOR normalized with respect to Tubulin. *p value less than 0.01 compared with previous group. (**C**) Tetramethylrhodamine, methyl ester (TMRM) dye accumulated in active mitochondria with intact potentials, was used to detect functional mitochondria in the MSCs at different ages following functional mitochondrial staining protocol from commercial. (**D**) Viability assay of mesenchymal stem cells from adult group incubated with 10 μM or 5 μM of imatinib mesylate or 1 ng/ml or 0.1 ng/ml of JK184. (**E**) Flow cytometry of Ki67 from mesenchymal stem cells of adult group incubated with 1 ng/ml or 0.1 ng/ml of JK184 in the medium. F) Western blot of mTOR and Tubulin of mesenchymal stem cells from adult group after incubating with 10 μM or 5 μM of imatinib mesylate or 1 ng/ml or 0.1 ng/ml of JK184 in the medium. Control = mesenchymal stem cells incubated with growth medium alone. The gels have been run under the same experimental conditions. *p value less than 0.01 compared with control group was considered statistically significant using Mann-Whitney-U tests.

All proteins from MSCs of rat bone marrow at different ages studied were compared between them. Summary each group was composed of a pool from 6 animals and two different iTRAQ experiments were performed. The results obtained in the iTRAQ study indicated that 1.072 proteins were identified, 201 of them statistically significant modulated between groups (Table 2). These proteins have been grouped by three processes attending String 9.0 software; those groups were proliferation (60 proteins), pluripotency (86 proteins) and energy metabolism (55 proteins) (Table 2). Significant activates pathways obtained by comparing modulated proteins obtained by iTRAQ analysis employing functional annotations according to the String 9.0 software and classified in three biological process for better comprehension were shown in Fig. 3. A. Several proteins found in our analysis associated with proliferation were 60Sribosomal proteins with different sedimentation speed like 60S RP L10, 60S RP L9, 60S RP L23, 60S RP L24, 60S RP L4, 60S RP L6 and 60S RP L7; also Vinculin which gene expression was validated by qRT-PCR analysis (Fig. 3D), all of them were statistically significant (p < 0.05) higher in newborn and adults with respect to the others groups. Superoxide dismutase-2 (SOD2) and Lamin A were increasing through the increasing age group like occurred in the iTRAQ analysis; all of them were validated by western blot (Fig. 3B). Mitosox and total ROS were studied by flow cytometry to explain SOD2 results in our iTRAQ study, ROS and mitosox were statistically significant (p < 0.05) lower in infant and pre-pubertal groups with respect to the others groups respectively (Fig. 4A,B). A fluorescence-based assay to detect functional mitochondria indicated that adult group had their functional mitochondria statistically significant decreased with respect to others groups of study (Fig. 5B). Proteins found in our iTRAQ analysis were associated with pluripotency like histones H1.5; H2B; H4 and protein disulfide-isomerase A1 (PDIA1) which were statistically significant (p < 0.05) high-regulated in infant and pubertal groups with respect to the others (Table 2). Several proteins found in our iTRAQ analysis associated whit energy metabolism were lactate dehydrogenase (LDH), glucose 6 phosphate dehydrogenase (G6PDH) and 6-phosphogluconate dehydrogenase (6PGDH), which were validated through analysis of their activity by enzimoimmunoassay (GPI). Lactate dehydrogenase (LDH) activity was increased in young group in front to infant and newborn group, decreased in pre-pubertal group in a statistically significant way (p < 0.01) and come back to increase its activity in pubertal and adult groups (Fig. 4C), glucose 6 phosphate dehydrogenase (G6PDH) and 6-phosphogluconate dehydrogenase (6PGDH) were statistically significant increased (p < 0.01) in pubertal and adult groups in front to others (Fig. 4D).

Immunoblot analysis indicated that mTOR and Raptor were statistically significant (p < 0.05) lower in pre-pubertal and pubertal groups with respect to the other groups studied. Adult group presented the statistically significant (p < 0.05) most increased level of mTOR and Raptor (Fig. 5A), the permanent dye TMRM analysis indicated a decrease in functional mitochondria which was statistically significant (p < 0.01) in infant and adult groups with respect to newborn and pubertal groups respectively (Fig. 5B). Viability assay using two physiological concentrations of Imatinib mesylate or JK184 did not affect the cells in culture (Fig. 5C). JK184 decreased statistically significant (p < 0.01) the expression of KI67 at 1mg/ml dose in culture (Fig. 5D). mTOR decreased dramatically in adult group when the cells were incubated with Imatinib mesylate or JK184 (Fig. 5E).

## Discussion

It is good known that long-term *in vitro* culture, but not chronological aging, alters their morphology, susceptibility to senescence and mitochondrial function. Thus, independent from donor animal age, *in vitro* aging of MSCs seems to result in complete loss of their progenitor characteristics^9^. Accordingly, functional analysis demonstrated altered mitochondrial morphology, decreased antioxidant capacities and elevated ROS levels in long-term cultivated independently the aged of the donor. Our present study is limited to the usage of rat MSCs instead can be able to apply on human MSCs; it provides direct comparison between chronological aged MSC not only at the cellular but also at the molecular level.

MSCs populations from different ageing groups were characterized by flow cytometry to check percentage of positive cells for mesenchymal markers, CD29 and CD90, and to check that were negative for hematopoietic markers (CD34, CD45) (Fig. 1A). We did not observe statistical significant differences between the mesenchymal markers into the different MSCs aging group studied (Fig. 2C). These were coincident with results published by Jin *et al.*^6^ indicating that MSCs have similar levels of surface antigen expression included MSCs from different tissues. Although the mesenchymal markers were not as abundant as published by Harting *et al.*^22^ these cells were able to adhere to the plastic plate and this is an intrinsic characteristic of mesenchymal stem cells and all of the groups were able to differentiate towards several mesoderm lineages (Fig. 2A,B). iTRAQ analysis is an adequate technique to study complex samples like we have used in this work^23^. Our results by iTRAQ analysis allowed the identification of 1.072 proteins. 201 of them were statistically significant modulated between groups (Table 2). Our study represents a step further from a previous iTRAQ-based study^24^ where 156 differentially expressed proteins were detected. Furthermore we compared six chronologically different groups, giving more strength to our study. To generate the quantitative proteome using iTRAQ labeling, it was first determined the labeling efficiency, which exceeded 99% (data not shown). Next, the cut-off for significant fold-change was determined based on the 2 biological replicates of two iTRAQ experiments which were chosen based on the following criteria: it contained more than 3 unique peptides (>95%) and p value < 0.05 for the 114/119 reporter ions. Accordingly, 90% of the commonly observed in the biological replicates fell within 25% of the respective experimental variation (Data don´t shown). The fold-change thresholds of >1.20 or <0.80 was set to identify true differences between expression of reporter ions.

One of the aims of our study was to establish the differences into proliferation process relating them to chronological donor age. Our results indicated that chronological age is directly influencing the expression of proliferation marker Ki67^25^ because of the lowest levels of Ki67 corresponded with less cells number in proliferation assays in infant and pre-pubertal groups. On the other way high levels of CD117, a self-renewal marker in MSCs as indicated Blazquez-Martinez *et al.*^26^ were corresponding to higher cells number in proliferation assay of pubertal and adult groups of animal respect to the rest groups (Fig. 1B,C).

60 modulated proteins found in our iTRAQ analysis were involved in proliferation as 60S ribosomal proteins with different sedimentation speed like 60S RP L10, 60S RP L9, 60S RP L23, 60S RP L24, 60S RP L4, 60S RP L6 and 60S RP L7 were over-expressed in young group respect to the others indicating their increased potential of pluripotency which would be in concordance with its low expression of Nanog gene by RT-PCR analysis (Fig. 2C) in this group as also Das *et al.*^27^ found over-expressed these proteins in different animal model process. Our results indicated that expression of Vinculin gene (Fig. 3C) was very low in newborn and young which were the most proliferative groups (Fig. 1B) on the opposite way pre-pubertal and adult presented high Vinculin gene expression coincidently with less proliferative potential. Toma-Jonik *et al.*^28^ published very recently that Vinculin, which is a protein involved in cell motility and adherence, was down-regulated in cells with great mobilization and proliferation potential like melanoma cells and at the same time Piltti *et al.*^29^ published that Rho kinase inhibitor (ROCKi) treatment increased the cellular proliferation up, in human foreskin fibroblast cells and, significantly less Vinculin-associated focal adhesions were present in these ROCKi-treated cells^30^. 11β-hydroxysteroid dehydrogenase type 1 (11β-HSD1), is an enzyme which generates glucocorticoids in intact cells, was found in our iTRAQ analysis significant increased in prepubertal group, this fact could explain the decrease proliferation potential and increase adypogenic differentiation in prepubertal group. All this is coincident with Bujalska *et al.*^31^ who published that 11β-HSD1 activity is uncommitted adipose stromal cells may facilitate proliferation rather than differentiation. Transforming growth factor β1 (TGF-β1) induces senescence in BM-MSCs via increases the mitochondrial reactive oxygen species production and also the ROS intracellular production is associated with decreasing mitochondrial membrane potential, DNA damage and cell senescence^32^,^33^, this fact could explain that statistically significant (p < 0.05) decreasing of total ROS in pre-pubertal group because TGF-β1 was found statistically significant (p < 0.05) low with respect to other groups in the iTRAQ analysis.

86 modulated proteins found in our iTRAQ analysis were involved in pluripotency process. Terme *et al.*^34^ showed that pluripotent cells had decreased levels of H1.0 and increased levels of H1.1, H1.3, and H1.5 compared with differentiated cells. Our results also indicated that H4 was statistically significant decrease in adult group which could point towards their less pluripotency with respect to the other groups, differentiation of embryonic stem cells is accompanied by a global reduction of panacetylation of histones H3 and H4 suggesting that histone acetylation plays an important role in maintenance of embryonic stem cells pluripotency^35^. Results published by Bermeo *et al.*^36^ indicate that MSCs over-expressing Lamin A had higher oestrogenic and lower radiogenic differentiation potential. Their studies demonstrated that lamin A/C played a significant role in the differentiation towards both osteoblast and adipocyte lines by regulating some of the elements of Wnt/β-catenin signaling during early MSCs differentiation, indicating that MSC over expressing Lamina A have higher osteogenic and lower adipogenic differentiation potential. Our results were coincident with Bermeo’s results, because of we found high levels of Lamina A/C by western in MSCs from adult group which we could link to lowest adipogenic potential with the statistically significant (p < 0.05) lowest levels of oil red staining during its directed differentiation (Fig. 2A,B). Also we consider the role of Lamin A like a senescence marker and its relationship whit increase of ROS^37^ together with increase of Thioredoxin found in our iTRAQ study in adult group could indicate the loss of functionality with age, it might be due to the accumulation of oxidative damage also induce because decrease of SOD2 in this adult group. Stolzing *et al.*^38^ demonstrated that age influences impairment of mesenchymal progenitor cells function.

55 modulated proteins found were involved in energetic metabolism process. The decision to exit pluripotency and undergo differentiation is of singular importance for pluripotent cells, including MSCs. The molecular mechanisms for these decisions to differentiate, as well as reversing those decisions during induced pluripotency have focused largely on transcriptomic controls. Easley *et al.*^39^ explored the role of translational control for the maintenance of pluripotency and the decisions to differentiate. ATP-citrate synthase is deep linked to pentose phosphate route and its inhibition has been recently linked to a decrease in proliferation rate^40^. Also it has been reported by Pattapa et cols.^41^ that MSCs resided under hypoxic conditions which were associated with the inherent metabolism of the cells. However MSCs under normoxia growth conditions derived a significant proportion of ATP from oxidative phosphorylation in addition to glycolysis. The observed increase of LDH in MSCs from adult group (Fig. 4C) could be explained because this group also had the glycolysis increased, on the other way glycolysis decreased in pre-pubertal group. All these results have been supported with our results through pentose phosphate pathway activity because of were significantly decreased G6PDH in pre-pubertal group and significantly increased in adult group (Fig. 4D).

Global protein translation is significantly reduced in hESCs compared to their differentiated progeny. mTOR is a Ser/Thr protein kinase that functions as an ATP and amino acid sensor to balance nutrient availability and cell growth^42^. mTOR regulates cellular senescence and drives bioenergetic infrastructure^12^. mTOR restrains proliferation potential of stem cells mediating their self-renewal loss, which is an effect that can be suppressed by mTOR-inhibitors, such as rapamicyn, antagonizing senescence^43^. mTOR plays an important role in the regulation of hematopoietic stem cell self-renewal *in vitro* and inhibition of mTOR hyperactivation with rapamycin may represent a novel approach to promote *ex vivo* expansion and their long-term hematopoietic reconstitution of hematopoietic stem cells^44^. Our results of mTOR family by western blot analysis indicated that mTOR (Fig. 5A,B) was statistically significant increased in adult group versus the other groups studied it could mean that MSCs from adult group were more senescence than MSCs from younger groups and this result was corroborated by expression of Lamin A/C in adult group in front at no expression of Lamin A in the other groups (Fig. 3B) together with less expression of TMRM (Fig. 5C) in adult group.

We found correlation between inhibition of mTOR and decrease of CD117 and Ki67 which are proliferation markers in the literature^45^,^46^ and we wonder if this relationship between mTOR pathway and proliferation was present when the proliferation markers were inhibited by specific reagents. Our results using inhibitors of CD117 and Ki67, imatinib mesylate and JK184 respectively, indicated that mTOR pathway can be modified through modification of proliferation markers, because the MSCs from adult group was treated with the Ki67 inhibitor at two physiological dose which do not affected their viability (Fig. 5D) but the expression of mTOR was modified when the proliferation marker Ki67 and CD117 were diminished (Fig. 5F).

## Conclusions

The importance of our study lies in the fact that age from the MSCs source directly influences their differentiation, proliferative and metabolism profiles and also it is the first time where is shown the direct influence of proliferative markers CD117 and Ki67 on activation of mTOR pathway. Summary we affirm that young group of rats has the most proliferative and pluripotent MSCs be able to future functional studies.

## Additional Information

**How to cite this article**: Fafián-Labora, J. *et al.* Influence of age on rat bone-marrow mesenchymal stem cells potential. *Sci. Rep.* **5**, 16765; doi: 10.1038/srep16765 (2015).

## Figures and Tables

**Table 1:** Specific primers for real-time reverse transcriptase-polymerase chain reaction (RT-PCR) amplification, listed with their annealing temperature (A.T.).

Gene Name	Fw primer	Rv primer	mRNA ID	A.T. (°C)
*Rex1*	gtgcatcacacctcagactgt	cgttggttgaaggccaactg	NM_005106.4	61
*Oct4*	ctcctggagggccaggaatc	atatacacaggccgatgtgg	NM_00510	61
*Sox2*	ctccgggacatgatcagc	ggtagtgctgggacatgtgaa	NM_001109181.1	61
*Nanog*	atgcctcacacggagactgt	aagtgggttgtttgcctttg	NM_005103.4	61
*Vinculin*	aggagaccttgcgaagacagg	gcggttgccacttgtttag	NM_001107248	61
*HPRT*	agccgaccggttctgtcat	agccgaccggttctgtca	NM_012583.2	61

Fw = forward; Rv = reverse.

**Table 2:** List of modulated proteins in mesenchymal stem cells at different ages classified according to their principal biological process using iTRAQ analysis.

Accession	**Name**	Peptides(95%)	**I/N**	**PVal I/N**	**Y/I**	**PVal Y/I**	**PP/Y**	**Pval PP/Y**	**P/PP**	**PVal P/PP**	**A/P**	**PVal A/P**
Metabolism												
Q6P783	6-phosphofructokinase	5	0,7228	0,1496	1,0497	0,7085	1,1027	0,3079	**1,4117**	**0,0388**	1,2082	0,2192
Q7TP11	6-phosphogluconate dehydrogenase, decarboxylating	5	0,8664	0,0404	0,9404	0,345	1,1936	0,1035	**1,2877**	**0,0026**	**0,6857**	**0,0099**
P06761	78 kDa glucose-regulated protein	35	0,9172	0,0089	0,9677	0,334	1,1997	0	0,8295	0,002	**1,4611**	**0**
M0RDC5	Acyl-CoA-binding protein (Fragment)	1	0,9923	0,9393	1,0172	0,8471	1,0129	0,8865	1,1744	0,4947	**1,408**	**0,0299**
F1LN88	Aldehyde dehydrogenase, mitochondrial	9	0,915	0,2981	0,9521	0,605	1,0255	0,8092	0,8105	0,1287	**1,249**	**0,0303**
P07943	Aldose reductase	9	1,0984	0,3717	0,8893	0,186	0,9549	0,5728	0,9715	0,7164	**1,2055**	**0,0389**
Q91W30	Aldose reductase-like protein	10	**1,6948**	**0,0054**	**0,5021**	**0,0183**	1,2079	0,2492	1,0106	0,9424	**1,2476**	**0,0229**
D3ZUM4	Beta-galactosidase	5	1,0315	0,7835	**1,3171**	**0,0441**	1,0942	0,385	1,1471	0,1506	0,9005	0,1892
O35567	Bifunctional purine biosynthesis protein PURH	13	0,9914	0,9389	0,8798	0,2663	1,1514	0,3918	0,8194	0,0166	**1,1312**	**0,0362**
Q99JD5	Branched-chain-amino-acid aminotransferase	5	1,0463	0,7187	0,842	0,0756	**1,3485**	**0,0127**	0,9146	0,4169	0,9297	0,5047
P15791	Calcium/calmodulin-dependent protein kinase type II subunit delta	5	0,9646	0,825	0,9676	0,8106	**1,3814**	**0,0071**	**0,7966**	**0,044**	1,0233	0,8049
G3V9E3	Caldesmon 1, isoform CRA_b	18	1,0648	0,1599	0,9241	0,1919	**1,4728**	**0**	**0,7388**	**0,0002**	**1,6353**	**0**
Q08290	Calponin-1	9	0,8714	0,0531	**1,3003**	**0,0464**	0,8214	0,2876	**0,723**	**0,0013**	**1,2572**	**0,0412**
P37397	Calponin-3	10	**1,3337**	**0,0378**	0,8281	0,0224	1,0857	0,2275	**0,7288**	**0,0035**	1,1632	0,1397
P18418	Calreticulin	12	**1,1514**	**0,0446**	0,8607	0,0394	**1,3403**	**0,0004**	**0,7119**	**0,0002**	**1,4109**	**0,0004**
G3V6S3	Calumenin	5	1,0591	0,4369	1,0768	0,3271	0,9833	0,8456	0,7673	0,2524	**1,5598**	**0,0141**
Q6P6T6	Cathepsin D	7	0,8801	0,1287	1,0622	0,3991	**1,5419**	**0,0105**	0,9111	0,1808	**1,3326**	**0,0234**
P97601	Chaperonin 10	3	1,0204	0,7796	1,0011	0,9902	0,9415	0,5177	0,939	0,4419	**1,4235**	**0,0087**
G3V936	Citrate synthase	3	**0,7934**	**0,0267**	1,167	0,0848	**0,7904**	**0,0267**	1,1452	0,2405	0,857	0,2318
F1M779	Clathrin heavy chain	9	**1,2157**	**0,0137**	1	0,9994	0,9612	0,7295	1,1343	0,2338	0,8853	0,3235
Q6TUH9	Corticosteroid 11-beta-dehydrogenase isozyme 1	3	0,7341	0,2686	1,3756	0,2277	**1,6421**	**0,0328**	1,9857	0,0672	0,7338	0,0642
P47875	Cysteine and glycine-rich protein 1	7	1,0015	0,9815	1,0448	0,5131	**1,2576**	**0,0488**	**0,6896**	**0,0389**	1,2501	0,0705
O08651	D-3-phosphoglycerate dehydrogenase	3	0,8786	0,1902	**0,7465**	**0,0308**	1,1981	0,1105	1,1565	0,1448	1,0565	0,5069
Q5BJ93	Enolase 1, (Alpha)	23	1,0226	0,655	1,026	0,5722	1,054	0,2406	**0,7412**	**0**	**1,2752**	**0,0123**
Q8R4A1	ERO1-like protein alpha	5	0,8236	0,0936	1,0042	0,9607	1,1541	0,1632	1,0604	0,6296	**1,4685**	**0,0439**
P05065	Fructose-bisphosphate aldolase A	7	**0,7852**	**0,0001**	0,9961	0,9275	1,1519	0,0055	1,17	0,0228	1,1494	0,0589
P11762	Galectin-1	13	0,8947	0,1869	1,0817	0,3224	0,9378	0,4491	**0,738**	**0,0127**	**1,2891**	**0,0226**
Q8CJG5	Gene	3	0,9562	0,7992	0,6552	0,233	**1,5338**	**0,0488**	1,0942	0,6762	0,9227	0,7939
P05370	Glucose-6-phosphate 1-dehydrogenase	16	1,0157	0,847	1,0285	0,7414	0,9947	0,9314	1,0827	0,1708	**1,2136**	**0,0003**
Q6P6V0	Glucose-6-phosphate isomerase	13	0,859	0,1021	0,9669	0,5321	1,1179	0,0929	1,1489	0,1394	**1,2446**	**0,0072**
P04797	Glyceraldehyde-3-phosphate dehydrogenase	28	1,0559	0,3012	**1,2268**	**0,0077**	0,8176	0,0404	1,0972	0,4988	0,8495	0,1048
P56574	Isocitrate dehydrogenase [NADP], mitochondrial	3	1,0223	0,8926	1,0131	0,8828	0,8721	0,3389	**1,3609**	**0,025**	0,8545	0,1231
B5DEN4	L-lactate dehydrogenase	14	**0,7058**	**0,0001**	1.146	0,00197	1.1828	0,0191	**1.2617**	**0**	**1.416**	**0**
Q6P7A9	Lysosomal alpha-glucosidase	4	0,9754	0,7869	1,0694	0,6226	**1,4268**	**0,048**	0,8687	0,254	1	1
Q6AYC4	Macrophage-capping protein	2	1,1578	0,3404	**1,3646**	**0,0086**	0,8334	0,049	0,7546	0,0948	1,3655	0,0617
F1LP60	Moesin (Fragment)	40	1,0168	0,6741	0,8227	0,0003	**1,4257**	**0**	0,9895	0,8299	0,9461	0,1782
P20070	NADH-cytochrome b5 reductase 3	2	0,7339	0,0716	**1,4911**	**0,0447**	0,9088	0,5256	0,9743	0,903	0,8315	0,169
Q6XD99	Non-erythroid spectrin beta	2	**1,3375**	**0,0053**	1,0624	0,4554	1,1109	0,3101	**0,7568**	**0,0122**	**1,5134**	**0,0041**
P16617	Phosphoglycerate kinase 1	29	**0,775**	**0**	1,0397	0,3579	1,0241	0,6829	1,1179	0,0446	**1,3076**	**0**
P25113	Phosphoglycerate mutase 1	9	**0,7559**	**0,0052**	1,1081	0,1211	1,1188	0,142	1,105	0,1848	**1,3205**	**0,0024**
P54001	Prolyl 4-hydroxylase subunit alpha-1	15	0,9432	0,3007	0,923	0,1417	**1,26**	**0,0005**	0,779	**0,0002**	0,9234	0,2253
M0R9D5	Protein Ahnak	60	**1,4134**	**0**	0,9734	0,2788	**1,2761**	**0**	0,8082	0	**2,1551**	**0**
D3ZIE9	Protein Aldh18a1	5	1,2134	0,1403	1,038	0,7002	**0,5751**	**0,0236**	0,8643	0,2248	0,7301	0,0622
M0R3 × 6	Protein LOC100912203	6	0,8645	0,163	1,0325	0,6321	1,1276	0,2248	0,9918	0,9068	**1,2992**	**0,0104**
D4A5L9	Protein LOC679794	4	**0,6761**	**0,0039**	**1,6157**	**0,0178**	**0,7517**	**0,0082**	0,9865	0,8661	1,0755	0,375
Q6P9U0	Protein Serpinb6	8	0,9886	0,8679	1,0239	0,6691	**1,3013**	**0,0066**	0,8823	0,2774	1,1196	0,16
D3ZF39	Protein Uap1	10	1,0277	0,7709	1,0072	0,947	**1,6544**	**0,0038**	0,9744	0,7756	**1,6238**	**0,0005**
B0BMT0	RCG47746, isoform CRA_a	90	1,0669	0,6161	**0,7141**	**0,0061**	**1,4858**	**0,0047**	**0,5813**	**0,0012**	0,7691	0,1418
Q6IRL3	Reticulon	7	1,0111	0,8671	0,8818	0,3109	1,3224	0,0677	**0,7406**	**0,0052**	0,9133	0,4439
B2GVB1	S100 calcium binding protein A6	3	1,0605	0,5609	1,2045	0,2186	**1,7118**	**0,0122**	**0,3918**	**0,0244**	**1,665**	**0,037**
Q5U3Z7	Serine hydroxymethyltransferase	3	**0,7739**	**0,0051**	0,9472	0,5152	1,0085	0,9511	1,1442	0,2328	1,0402	0,831
F1M953	Stress-70 protein, mitochondrial	12	0,9377	0,2437	**0,7924**	**0,0057**	1,174	0,0025	0,8961	0,1199	**1,5666**	**0**
P48500	Triosephosphate isomerase	16	**0,6753**	**0,0004**	**1,174**	**0,0315**	1,0854	0,3958	1,2639	0,1091	**1,4017**	**0,0001**
Q9Z1A6	Vigilin	3	1,1784	0,0874	0,8426	0,0918	1,0244	0,862	0,9978	0,9727	**1,2104**	**0,0345**
P81155	Voltage-dependent anion-selective channel protein 2	6	1,0012	0,9931	1,0633	0,4458	**1,2708**	**0,0343**	0,8377	0,1616	0,992	0,9444
Pluripotency												
P63102	14-3-3 protein zeta/delta	24	0,9971	0,9573	0,9743	0,6297	1,0748	0,2065	0,8947	0,1104	**1,1562**	**0,0250**
Q7TP91	Ab1-205	3	0,9802	0,8434	1,2607	0,1387	0,9026	0,3795	1,0629	0,7975	**0,6232**	**0,0302**
Q64640	Adenosine kinase	2	0,9609	0,8197	1,1207	0,3134	1,1155	0,346	1,0828	0,4536	**0,6855**	**0,0454**
P39069	Adenylate kinase isoenzyme 1	4	0,7781	0,0775	1,2606	0,053	1,0747	0,7679	0,9795	0,8759	**1,6777**	**0,029**
P23928	Alpha-crystallin B chain	5	1,207	0,0573	**1,8689**	**0,0119**	**2,2257**	**0,0003**	**0,2931**	**0,0035**	1,5629	0,0723
Q6IMZ3	Annexin A6	24	**1,2484**	**0,0001**	1,1079	0,0199	0,9791	0,5623	1,0031	0,9354	1,0187	0,5863
Q07936	Annexin A2	22	**1,2928**	**0**	1,0234	0,6825	**1,368**	**0,0001**	0,8172	0,003	1,072	0,1567
Q05175	Brain acid soluble protein 1	3	0,9481	0,8617	1,209	0,454	1,3727	0,148	**0,4584**	**0,0445**	1,6462	0,1009
Q6T487	Brain-specific alpha actinin 1 isoform	48	**0,7786**	**0,0007**	1,1584	0,0465	1,0249	0,6506	0,8259	0,0018	1,1052	0,2188
Q8R4A2	Caveolin 1 (Fragment)	4	0,9676	0,8582	**1,8465**	**0,0328**	1,0661	0,7396	0,7326	0,1573	1,0726	0,6061
P02454	Collagen alpha-1(I) chain	21	**1,9314**	**0,0008**	**0,4642**	**0**	**1,3783**	**0**	1,1251	0,0342	1,123	0,1023
F1LS40	Collagen alpha-2(I) chain	19	**1,486**	**0,0008**	**0,6971**	**0**	1,1467	0,0083	0,9555	0,2908	**1,3211**	**0,0003**
P07335	Creatine kinase B-type	3	**0,7036**	**0,0109**	1,1785	0,2497	1,4259	0,0546	0,8379	0,2386	1,2443	0,3604
F1LMA7	C-type mannose receptor 2	5	1,1259	0,191	0,8731	0,3184	0,8322	0,3836	0,8408	0,3689	**1,7697**	**0,0009**
P47875	Cysteine and glycine-rich protein 1	5	1,0995	0,2349	0,9264	0,4028	**1,275**	**0,0227**	**0,7329**	**0,0396**	1,2025	0,1773
Q6AYI1	DEAD (Asp-Glu-Ala-Asp) box polypeptide 5	9	0,9335	0,2865	0,9937	0,9257	0,8965	0,0659	**1,2504**	**0,0032**	0,9596	0,434
Q62952	Dihydropyrimidinase-related protein 3	8	**1,2443**	**0,0364**	**1,5656**	**0,0028**	0,858	0,2853	0,8247	0,0165	1,0088	0,9184
Q4V8H8	EH domain-containing protein 2	0	0,9656	0,7399	1,3396	0,2041	0,8004	0,248	**1,6703**	**0,0471**	0,7495	0,491
Q68FR6	Elongation factor 1-gamma	9	1,0087	0,8562	0,9072	0,4157	**1,2134**	**0,0011**	0,9535	0,5667	0,8485	0,0109
C0JPT7	Filamin alpha	100	**1,2134**	**0**	**1,3022**	**0**	0,8202	0	0,9147	0,0068	1,386	0
D4A8D5	Filamin, beta (Predicted)	19	1,0951	0,0867	**1,2541**	**0,0012**	0,8193	0,0061	**0,7937**	**0,0124**	1,329	0,0009
B6DYQ7	Glutathione S-transferase pi	4	1,0946	0,3206	1,1008	0,6055	**1,5111**	**0,0241**	**3,1507**	**0,0004**	**0,3406**	**0,0033**
G3V913	Heat shock 27kDa protein 1	5	1,6407	0,0562	0,9647	0,8462	**1,5674**	**0,0118**	**0,636**	**0,038**	**1,3145**	**0,0077**
P63018	Heat shock cognate 71 kDa protein	30	0,9815	0,6625	**1,2334**	**0,0221**	0,8841	0,0143	1,0164	0,8526	1,1903	0,0184
F1M3D3	Heterogeneous nuclear ribonucleoprotein M	3	**0,6983**	**0,0017**	1,0334	0,5966	0,9158	0,4502	1,1725	0,1239	1,0388	0,7714
Q6IMY8	Heterogeneous nuclear ribonucleoprotein U	8	**0,7965**	**0,0032**	1,0228	0,7409	0,9994	0,9956	1,1924	0,0262	0,8534	0,1414
P15865	Histone H1.4	8	**0,7411**	**0,0048**	**2,4013**	**0,0005**	1,0711	0,2301	**0,5016**	**0,0007**	**0,6377**	**0,007**
D3ZBN0	Histone H1.5	4	**1,911**	**0,0166**	**0,4907**	**0,0135**	0,9905	0,9189	1,4031	0,0552	0,8838	0,2767
G3V9C7	Histone H2B	20	**1,2535**	**0,0051**	0,9957	0,9764	0,8311	0,1792	**1,4957**	**0,0005**	**0,7706**	**0,0027**
M0RBX6	Histone H3	6	1,1323	0,058	**1,4413**	**0,0125**	**1,201**	**0,0272**	**0,5991**	**0,0004**	**0,494**	**0,0002**
P62804	Histone H4	13	**1,4819**	**0,0057**	**0,5999**	**0,0004**	1,1515	0,0641	**1,7155**	**0,0008**	**0,6785**	**0,002**
Q6P6G9	Hnrpa1 protein	8	**0,6091**	**0,0304**	0,9626	0,6587	0,943	0,663	1,1142	0,7189	0,969	0,7865
P50503	Hsc70-interacting protein	4	0,9785	0,7614	1,0509	0,7519	1,1426	0,4415	0,8392	0,2581	**1,5699**	**0,0074**
P49134	Integrin beta-1	6	**1,5471**	**0,0002**	0,9023	0,1179	**1,5398**	**0,0098**	**0,7217**	**0,0064**	**1,311**	**0,0037**
G3V7Q7	IQ motif containing GTPase activating protein 1 (Predicted), isoform CRA_b	29	0,9058	0,0331	0,8665	0,0016	**1,2223**	**0**	1,0398	0,2744	0,8961	0,0032
Q6TXE9	LRRGT00050	4	0,8206	0,0425	0,8878	0,4999	1,0972	0,6832	**1,5**	**0,0036**	0,8044	0,129
Q6TUD1	LRRGT00113	2	**0,7362**	**0,0289**	1,0159	0,9238	1,0387	0,7991	1,0836	0,5779	0,9606	0,8364
Q5M7W5	Microtubule-associated protein 4	2	1,6111	0,1601	0,845	0,5023	1,0907	0,433	0,6693	0,1236	**1,6243**	**0,0414**
B2GV99	Myl6 protein	11	1,0049	0,9535	1,1269	0,1948	1,0655	0,29	0,9193	0,1676	**1,3394**	**0,0011**
G3V9Y1	Myosin, heavy polypeptide 10, non-muscle, isoform CRA_b	51	0,9356	0,0771	1,0793	0,1827	0,8786	0,0186	0,8988	0,0205	**0,792**	**0,0018**
G3V6P7	Myosin, heavy polypeptide 9, non-muscle	98	0,9405	0,0071	1,1877	0	1,0117	0,6464	0,957	0,2338	**1,3299**	**0**
P05982	NAD(P)H dehydrogenase [quinone] 1	8	**1,3454**	**0,0036**	**0,7746**	**0,0108**	1,2457	0,0611	0,817	0,2198	**1,7303**	**0,0017**
G3V8R1	Nucleobindin 2, isoform CRA_b	3	**0,7234**	**0,0259**	**2,0743**	**0,0023**	**0,5945**	**0,0061**	0,8133	0,1039	**1,5101**	**0,0176**
F1M4W3	Palladin (Fragment)	6	1,0033	0,9633	0,8726	0,1096	1,0418	0,6509	**0,6823**	**0,0117**	1,0929	0,2707
P52944	PDZ and LIM domain protein 1	8	1,0741	0,3026	1,0802	0,1599	**1,3743**	**0,0005**	0,9648	0,7456	**1,2349**	**0,03**
Q62920	PDZ and LIM domain protein 5	17	0,9467	0,5156	**0,6947**	**0,0022**	**1,4784**	**0,0057**	**0,6885**	**0,0358**	0,8014	0,071
Q6AYQ9	Peptidyl-prolyl cis-trans isomerase	6	0,9218	0,2069	0,9782	0,6989	1,2033	0,0649	**0,7408**	**0,0209**	0,837	0,0475
Q62658	Peptidyl-prolyl cis-trans isomerase FKBP1A	2	1,254	0,0638	1,1087	0,2151	1,0291	0,6981	0,8516	0,0786	**1,38**	**0,0149**
D3ZAF5	Periostin, osteoblast specific factor (Predicted), isoform CRA_a	4	0,5315	0,1266	1,4489	0,0583	**0,7663**	**0,0352**	1,3251	0,0507	0,8907	0,4068
Q63716	Peroxiredoxin-1	13	0,8935	0,0404	1,0622	0,5224	1,083	0,5586	0,884	0,3841	**1,2919**	**0,0335**
P35704	Peroxiredoxin-2	5	0,9438	0,6729	**1,3399**	**0,0331**	0,9268	0,3898	0,8375	0,2252	1,1003	0,4352
Q9R063	Peroxiredoxin-5, mitochondrial	5	1,0571	0,6808	0,7826	0,0577	1,2214	0,0908	0,8798	0,195	**1,4277**	**0,0498**
F1LPK7	Phospholipid scramblase 3	5	**1,3539**	**0,015**	**0,7564**	**0,0206**	**1,2564**	**0,0285**	0,9783	0,8008	1,0061	0,9704
G3V8L9	Polymerase I and transcript release factor	10	1,0181	0,7755	**1,5741**	**0,0001**	**1,2442**	**0,0026**	**0,6574**	**0,0001**	1,2032	0,1746
G3V9I0	Procollagen-lysine,2-oxoglutarate 5-dioxygenase 2	15	**0,6772**	**0,0001**	**1,3393**	**0,0139**	0,8435	0,0459	1,1106	0,2824	1,033	0,765
D3ZRX9	Protein Cnn2	9	0,9803	0,7232	0,9782	0,6955	1,1542	0,0382	**0,7727**	**0,0037**	1,1053	0,1116
G3V6T7	Protein disulfide isomerase associated 4	4	1,094	0,3778	**1,5291**	**0,0042**	0,7583	0,087	0,8527	0,0368	1,1596	0,0466
P04785	Protein disulfide-isomerase	18	0,9524	0,2161	0,9019	0,0205	1,1449	0,002	0,91	0,0672	**1,3562**	**0**
P11598	Protein disulfide-isomerase A3	23	1,0044	0,9331	1,1813	0,0003	1,0096	0,899	0,888	0,1863	1,151	0,1114
Q63081	Protein disulfide-isomerase A6	9	**0,7832**	**0,0043**	1,1044	0,262	**1,2788**	**0,0234**	0,9335	0,4195	1,0394	0,5727
D3ZHA0	Protein Flnc	28	0,9537	0,2739	**1,6131**	**0**	0,8375	0,0128	0,9354	0,1802	1,1796	0,0125
E2RUH2	Protein LOC100360501	3	0,8715	0,4837	**1,2547**	**0,0379**	**0,7763**	**0,024**	1,2418	0,1487	**0,7978**	**0,0359**
M0R7B4	Protein LOC684828	6	**1,9171**	**0,003**	**0,4959**	**0,0035**	1,0813	0,3542	**1,3227**	**0,0305**	0,8447	0,1021
F1MA29	Protein LOC685520	5	**0,7506**	**0,0026**	1,15	0,0892	0,9181	0,414	1,0561	0,438	1,066	0,4416
D3ZUB0	Protein Rcn1	2	1,0185	0,8167	0,8941	0,2217	1,1262	0,2053	0,8607	0,297	**1,3273**	**0,0313**
I6L9G5	Protein Rcn3	2	1,0873	0,4834	**0,5646**	**0,023**	1,147	0,2936	0,9716	0,7715	1,3833	0,2381
D4A1P2	Protein Rpl10l	7	1,0101	0,8587	0,8912	0,0814	0,8855	0,0554	**1,4153**	**0,0002**	**0,6894**	**0,0001**
F1M853	Protein Rrbp1	12	0,9865	0,8266	0,9487	0,2254	**1,4058**	**0,0002**	**0,6416**	**0,0002**	**1,4396**	**0**
P05942	Protein S100-A4	8	1,3344	0,0883	0,8596	0,3432	**2,371**	**0,0005**	**0,6816**	**0,0449**	**1,6256**	**0,0034**
B0BMT9	Protein Sqrdl	5	0,8772	0,1123	0,8745	0,2138	**1,3482**	**0,0318**	**0,672**	**0,0272**	1,1637	0,2917
P50399	Rab GDP dissociation inhibitor beta	5	**0,6527**	**0**	1,1226	0,1842	0,8087	0,0191	1,2167	0,0909	0,8327	0,0497
Q5FVG5	Similar to tropomyosin 1, embryonic fibroblast-rat, isoform CRA_c	21	0,8189	0,0635	0,8236	0,0664	**1,5332**	**0,0055**	**0,4284**	**0,0029**	0,9622	0,622
Q6IRH6	Slc25a3 protein	5	**0,6221**	**0,0046**	1,2418	0,0813	0,9018	0,3869	**1,2937**	**0,0271**	**0,7334**	**0,0095**
P06685	Sodium/potassium-transporting ATPase subunit alpha-1	6	1,0612	0,5229	0,847	0,0206	0,9521	0,6531	**1,1204**	**0,4092**	**0,8973**	**0,1495**
P16975	SPARC	5	1,2574	0,0585	0,8963	0,1879	1,0501	0,5303	0,9358	0,5629	**1,2361**	**0,0437**
Q63413	Spliceosome RNA helicase Ddx39b	4	**0,7567**	**0,0206**	1,0276	0,7991	0,904	0,431	**1,3219**	**0,0189**	0,679	0,0523
Q6IRK8	Spna2 protein	9	**1,3867**	**0**	**1,211**	**0,003**	1,0232	0,7136	**0,7877**	**0,0016**	**1,7205**	**0**
D4A8Y5	Staphylococcal nuclease domain-containing protein 1	3	0,919	0,451	**1,2908**	**0,0445**	0,8256	0,0815	1,2376	0,0644	0,7802	0,1202
Q71SA3	Thrombospondin 1	7	0,8058	0,0398	**0,7166**	**0,0007**	**1,3192**	**0,0007**	0,9713	0,6767	**1,4974**	**0,0023**
P31232	Transgelin	39	**1,2096**	**0,0003**	1,2967	0,0743	1,133	0,0136	**0,5637**	**0,0001**	**1,7372**	**0**
Q5XFX0	Transgelin-2	17	0,9888	0,8414	1,0039	0,955	**1,3666**	**0,0009**	0,9786	0,7678	1,1396	0,048
Q6AYT3	tRNA-splicing ligase RtcB homolog	4	**0,6896**	**0,019**	0,9867	0,8815	0,8815	0,1894	1,1372	0,1808	0,8525	0,4565
Q63610	Tropomyosin alpha-3 chain	9	0,9843	0,838	**1,4987**	**0,0199**	1,2255	0,0709	0,6776	0,0538	**1,8819**	**0,0153**
P09495	Tropomyosin alpha-4 chain	12	0,9739	0,7802	1,356	0,0682	1,0117	0,925	0,8335	0,1905	**1,5771**	**0,0401**
G3V6C4	UDP-glucose 6-dehydrogenase	8	1,0597	0,478	0,9444	0,6809	**1,256**	**0,011**	1,0009	0,9911	**1,4008**	**0,0135**
Q63355	Unconventional myosin-Ic	10	**1,2333**	**0,0007**	0,8095	0,0071	**1,2252**	**0,0431**	1,0652	0,3451	0,8854	0,0253
P31000	Vimentin	110	1,0703	0,0545	1,0991	0,0394	1,1756	0,0018	**0,76**	**0**	0,9955	0,9047
Proliferation												
P62268	40S ribosomal protein S23	3	1,0729	0,3955	**0,7949**	**0,0312**	1,0116	0,8769	0,9447	0,6609	1,0981	0,4568
M0RD75	40S ribosomal protein S6 (Fragment)	5	**1,213**	**0,0348**	**0,7478**	**0,0343**	1,0648	0,6105	0,8839	0,3222	1,2172	0,0637
B2RYR8	40S ribosomal protein S8	5	1,156	0,0743	**0,7651**	**0,0113**	0,9563	0,6408	1,1034	0,3318	1,0474	0,6037
P29314	40S ribosomal protein S9	10	**1,2232**	**0,0078**	**0,7297**	**0,0002**	1,1604	0,014	1,0442	0,4378	1,0231	0,6427
P38983	40S ribosomal protein SA	7	1,0045	0,948	0,8267	0,0864	1,0707	0,3666	**1,3083**	**0,0159**	**0,8177**	**0,0379**
P63039	60 kDa heat shock protein, mitochondrial	14	1,0284	0,7397	1,0424	0,6331	1,0209	0,827	**0,7502**	**0,0126**	1,1048	0,3793
Q6PDV7	60S ribosomal protein L10	8	**1,2944**	**0,0232**	0,7476	0,0503	1,0811	0,4911	0,9186	0,3735	1,079	0,2893
P41123	60S ribosomal protein L13	4	1,2788	0,1225	0,8351	0,0607	1,0613	0,7175	**1,269**	**0,0179**	0,8472	0,0781
P61314	60S ribosomal protein L15	2	1,0608	0,6431	0,9332	0,5211	0,8939	0,228	**1,6856**	**0,0065**	**0,7429**	**0,0338**
Q0QEW8	60S ribosomal protein L18 (Fragment)	3	0,8899	0,3903	1,0505	0,6391	0,9184	0,5621	**1,4963**	**0,0365**	0,7225	0,0735
P62718	60S ribosomal protein L18a	4	1,0118	0,8521	0,8707	0,2832	1,0157	0,9288	**1,3147**	**0,032**	0,8694	0,1711
P62832	60S ribosomal protein L23	6	**1,2256**	**0,0212**	**0,838**	**0,0334**	0,9969	0,9615	0,9723	0,8064	1,1553	0,1309
P83732	60S ribosomal protein L24	7	**1,5524**	**0,0024**	**0,5064**	**0,0004**	**1,2108**	**0,0391**	0,8698	0,2155	**1,2834**	**0,0175**
P25886	60S ribosomal protein L29	3	**1,5331**	**0,0443**	0,9651	0,7362	1,0984	0,4167	0,8404	0,5923	0,7419	0,2733
P21531	60S ribosomal protein L3	5	1,0552	0,6436	0,8494	0,0879	0,932	0,5476	**1,463**	**0,0213**	**0,7223**	**0,0148**
Q6P3V9	60S ribosomal protein L4	9	**1,419**	**0,0021**	**0,627**	**0,0002**	1,0227	0,7849	0,9688	0,6057	1,139	0,0543
P09895	60S ribosomal protein L5	6	0,9424	0,3628	0,9732	0,6933	0,9466	0,5073	**1,2345**	**0,0225**	0,8597	0,1305
H7C5Y5	60S ribosomal protein L6	7	**1,3496**	**0,0261**	**0,6716**	**0,0068**	1,0461	0,66	1,0542	0,451	1,0501	0,6358
Q6P790	60S ribosomal protein L6 (Fragment)	7	**1,2191**	**0,0025**	1,0302	0,7281	1,0286	0,6572	0,8965	0,2512	0,987	0,8763
P05426	60S ribosomal protein L7	5	**1,3498**	**0,0462**	0,6788	0,065	1,1405	0,3092	0,9309	0,3885	1,0532	0,5387
P85970	Actin-related protein 2/3 complex subunit 2	11	0,9564	0,6557	0,9226	0,2715	**1,3327**	**0,0061**	1,0044	0,938	1,0774	0,3839
Q9Z1P2	Alpha-actinin-1	77	0,8727	0,0028	0,8694	0,0002	**1,3424**	**0,0013**	0,8164	0,0001	0,9543	0,1358
Q9QXQ0	Alpha-actinin-4	50	**1,2074**	**0,0004**	1,1204	0,037	1,0349	0,3567	0,9069	0,2105	1,1419	0,036
Q66HH8	Annexin 5	9	0,9925	0,8998	1,0383	0,7603	0,9707	0,811	0,9909	0,8915	**1,4149**	**0,0034**
P45592	Cofilin-1	12	**1,2442**	**0,0041**	1,0603	0,5475	1,0804	0,2025	0,9203	0,5481	**1,2685**	**0,011**
D3ZH41	Cytoskeleton-associated protein 4 (Predicted)	12	**0,7853**	**0,0004**	1,107	0,0513	0,9476	0,3458	**0,7919**	**0,0005**	**1,2344**	**0,0033**
Q6AYH5	Dynactin subunit 2	3	1,151	0,2739	0,8845	0,3112	1,1661	0,2508	0,8114	0,3778	**1,3787**	**0,0248**
P52555	Endoplasmic reticulum resident protein 29	2	1,0186	0,8459	1,3064	0,1141	1,2519	0,2488	0,8626	0,2226	**1,5742**	**0,0319**
Q6P3V8	Eukaryotic translation initiation factor 4A1	13	1,0465	0,4461	0,8673	0,0328	1,055	0,416	1,0269	0,6429	**0,7893**	**0,0001**
P04937	Fibronectin	17	1,1267	0,3104	**0,7887**	**0,0119**	**1,2538**	**0,0001**	0,6481	0,0907	**2,4985**	**0,0056**
Q6P792	Four and a half LIM domains 1	6	**0,7431**	**0,0006**	**1,7903**	**0,0001**	1,1108	0,096	**0,7768**	**0,0085**	0,8731	0,0375
P11762	Galectin-1 OS=Rattus norvegicus	14	**0,7674**	**0,0204**	0,7313	0,053	1,2804	0,0564	0,8213	0,4313	1,1116	0,227
B6DYQ2	Glutathione S-transferase mu 2	5	1,1023	0,4484	1,0507	0,6202	0,8053	0,0678	0,8616	0,3082	**1,2838**	**0,0235**
P63245	Guanine nucleotide-binding protein subunit beta-2-like 1	6	1,0188	0,7481	0,8543	0,044	1,0255	0,6962	1,0046	0,963	0,9843	0,8938
Q6P7Q4	Lactoylglutathione lyase	6	0,8942	0,2376	0,9113	0,2336	**1,2099**	**0,0449**	0,9186	0,2639	1,0132	0,8509
G3V8L3	Lamin A, isoform CRA_b	26	0,9048	0,0029	0,968	0,4478	1,1812	0,0001	1,0126	0,746	**1,2067**	**0**
Q99MZ8	LIM and SH3 domain protein 1	5	**1,4773**	**0,0032**	1,0103	0,9316	1,1251	0,1144	**0,7622**	**0,0071**	**1,3922**	**0,0031**
O08557	N(G), N(G)-dimethylarginine dimethylaminohydrolase 1	4	0,8682	0,4556	**2,2145**	**0,0173**	**0,5358**	**0,0395**	0,7504	0,1763	**1,2909**	**0,0453**
Q6S3A0	Plectin 6	28	1,0437	0,2665	0,9378	0,1139	1,1564	0,0026	0,996	0,9259	**1,2269**	**0,0027**
D4A4Z9	Protein Ktn1	7	0,9773	0,8388	0,901	0,2289	**1,3423**	**0,0487**	**0,7373**	**0,0131**	1,0952	0,3487
D3ZPL5	Protein LOC100361311	10	**1,2843**	**0,0356**	**0,683**	**0,017**	1,0148	0,8697	0,9663	0,7274	1,1144	0,0623
M0RCY2	Protein LOC683961	6	0,9886	0,9133	0,9596	0,6555	**0,761**	**0,0353**	**1,4335**	**0,0202**	**0,591**	**0,0003**
D3ZN21	Protein RGD1309586	6	0,951	0,6075	0,9746	0,7735	0,957	0,6381	**1,2502**	**0,0226**	0,8539	0,0205
D4A6W6	Protein RGD1561333	6	**1,7423**	**0,0038**	**0,5049**	**0,0038**	1,2265	0,2464	0,8945	0,5405	1,1717	0,2285
D4A6W6	Protein RGD1561333	5	1,1352	0,1387	1,0601	0,5478	0,9173	0,2914	**1,5332**	**0,0034**	**0,6202**	**0,0124**
F1LT35	Protein RGD1564606 (Fragment)	6	1,1172	0,1379	1,1037	0,4799	1,0681	0,4407	**0,772**	**0,0124**	**1,2544**	**0,0157**
G3V852	Protein Tln1	38	**1,2686**	**0**	**1,2229**	**0**	0,8272	0	0,9642	0,3307	**1,3768**	**0**
Q4QQV0	Protein Tubb6	22	1,0776	0,4062	0,8668	0,3417	**1,2995**	**0,0298**	0,946	0,6332	1,0638	0,6569
Q6P3E1	Rps16 protein (Fragment)	7	**1,6881**	**0,0047**	**0,5471**	**0,0061**	**1,508**	**0,0481**	0,8378	0,1083	1,2384	0,0629
Q9QZR6	Septin-9	6	1,0595	0,4348	0,9334	0,3019	**1,2185**	**0,0277**	0,8772	0,1483	1,017	0,8438
Q6LDS4	Superoxide dismutase [Cu-Zn]	6	1,1464	0,0737	1,169	0,0483	0,8679	0,0643	1,0068	0,9549	**1,3603**	**0,0094**
P07895	Superoxide dismutase [Mn], mitochondrial	10	0,76	0,2573	0,9976	0,9829	**2,2825**	**0,02**	1,074	0,2946	**1,3438**	**0,0046**
P28480	T-complex protein 1 subunit alpha	6	0,7782	0,0579	**1,5251**	**0,0086**	0,9731	0,7177	1,2381	0,2501	0,9675	0,8659
Q68FQ0	T-complex protein 1 subunit epsilon	4	0,8933	0,2273	1,0047	0,9576	0,9601	0,7021	1,1826	0,1497	0,8175	0,0229
Q6P502	T-complex protein 1 subunit gamma	5	1,0456	0,4189	0,8549	0,0333	1,0502	0,74	1,0936	0,3134	0,9009	0,3475
P11232	Thioredoxin	9	0,9519	0,7004	0,9806	0,8828	1,1098	0,4942	0,9058	0,4438	**1,2619**	**0,0373**
Q99PD6	Transforming growth factor beta-1-induced transcript 1 protein	6	1,0858	0,7029	0,7535	0,8138	1,2703	0,2603	**0,635**	**0,0115**	0,8607	0,8969
P68370	Tubulin alpha-1A chain OS=Rattus norvegicus GN=Tuba1a PE=1 SV=1	19	1,1996	0,0477	1,1154	0,0721	0,7776	0,0946	0,9411	0,7092	**0,7696**	**0,0004**
R9PXU6	Vinculin	57	1,1908	0	0,9981	0,9534	**1,4169**	**0**	0,8383	0,0001	1,1453	0
